# Dr. John S. Billings, U. S. Army

**Published:** 1895-10

**Authors:** 


### Dr. John S. Billings, U. S. Army.

*L'Union Medicale* of August 31st, says: "Surgeon-General Billings, of the United States Army, has asked to be retired in the month of October. This very distinguished confrere will leave the Army Medical Museum, of which he is administrator, and the library of the Surgeon-General's office, of which he is librarian, institutions which have been made what they are by his ability and devotion.

"Before taking his retirement Surgeon-General Billings hopes to terminate the last volume of the Index Catalogue, an immense and precious work for which the entire medical profession should be grateful to the Government of the United States, its Army Medical Department, and above all to Dr. John Shaw Billings. In retirement Dr. Billings will not cease to work. He has accepted the Chair of Hygiene at the University of Pennsylvania."

The final volume of the Index Catalogue has just come to hand, and is in all respect equal to its predecessors. This series of sixteen large volumes of index matter, gathered from the entire field of medical literature, and is of incalculable value.

